# Synergistic Effects of Caffeine in Combination with Conventional Drugs: Perspectives of a Drug That Never Ages

**DOI:** 10.3390/ph16050730

**Published:** 2023-05-11

**Authors:** Davide Ialongo, Valeria Tudino, Merve Arpacioglu, Antonella Messore, Elisa Patacchini, Roberta Costi, Roberto Di Santo, Valentina Noemi Madia

**Affiliations:** Istituto Pasteur-Fondazione Cenci Bolognetti, Dipartimento di Chimica e Tecnologie del Farmaco, Sapienza Università di Roma, p.le Aldo Moro 5, I-00185 Rome, Italy; davide.ialongo@uniroma1.it (D.I.); valeria.tudino@gmail.com (V.T.); merve.arpacioglu@uniroma1.it (M.A.); antonella.messore@uniroma1.it (A.M.); elisa.patacchini@uniroma1.it (E.P.); roberta.costi@uniroma1.it (R.C.); roberto.disanto@uniroma1.it (R.D.S.)

**Keywords:** caffeine, synergism, multidrug therapy, anti-inflammatory drugs, anticancer drugs, antimicrobial drugs, opioids, neurodegenerative diseases, antidiabetic drugs, anti-obesity drugs

## Abstract

Plants have been known since ancient times for their healing properties, being used as preparations against human diseases of different etiologies. More recently, natural products have been studied and characterized, isolating the phytochemicals responsible for their bioactivity. Most certainly, there are currently numerous active compounds extracted from plants and used as drugs, dietary supplements, or sources of bioactive molecules that are useful in modern drug discovery. Furthermore, phytotherapeutics can modulate the clinical effects of co-administered conventional drugs. In the last few decades, the interest has increased even more in studying the positive synergistic effects between plant-derived bioactives and conventional drugs. Indeed, synergism is a process where multiple compounds act together to exert a merged effect that is greater than that of each of them summed together. The synergistic effects between phytotherapeutics and conventional drugs have been described in different therapeutic areas, and many drugs are based on synergistic interactions with plant derivatives. Among them, caffeine has shown positive synergistic effects with different conventional drugs. Indeed, in addition to their multiple pharmacological activities, a growing body of evidence highlights the synergistic effects of caffeine with different conventional drugs in various therapeutic fields. This review aims to provide an overview of the synergistic therapeutic effects of caffeine and conventional drugs, summarizing the progress reported to date.

## 1. Introduction

Multidrug therapy is a powerful plan relying on the blocking or elimination of harmful factors (e.g., cancer cells or pathogens) together with activating the body’s defenses or repair processes. It arises from a gradual shift from the principle of mono-substance therapy (i.e., using a single active principle)—on which the pharmaceutical research has been based for decades—towards multidrug therapy with drug combinations [1]. More broadly, the term multidrug therapy can refer not only to the use of two or more drugs for a single therapeutic indication, but also to the administration of two or more drugs to treat two or more chronic pathological conditions (i.e., multimorbidity) in an individual. Notably, an increased prevalence of patients with multimorbidity—who are more likely to receive multidrug therapy—has been observed in recent years because of longer life expectancies [1]. Indeed, for the treatment of multifactorial or complex diseases (e.g., diabetes and other metabolic diseases, cancer, hypertension, acquired immune deficiency syndrome (AIDS), malaria, tuberculosis), multidrug therapy is commonly adopted [2].

The rationale behind the multidrug strategy relies on the knowledge that many diseases have a multifaceted etiology and/or complications that can be targeted better with drug combinations than with a single administration, while also promoting patient compliance. Furthermore, multidrug therapy is characterized by better efficacy and diminished toxicity and/or drug resistance, making it a standard of treatment for several diseases and a promising approach for unmet clinical needs such as Alzheimer’s disease [3].

The progressive move from the long-established mono-drug therapy to the newer idea of multidrug therapy is considerably promoting phytotherapy [4]. Indeed, phytotherapy plays a key “innate” role, given that it relies on the effectiveness of herbs or plants, which contain complex mixtures of bioactive compounds that complement one another to achieve greater efficacy. Furthermore, compounds of natural origin have several advantages, e.g., they are cheaper, they come from renewable sources, and they are sources of new chemical scaffolds for drug development (with about one-third of drugs administered in clinics developed from plants). For instance, tetracycline, artemisinin, doxorubicin, vinca alkaloids, rohitukine, morphine and its derivatives, quinine and quinidine, penicillin and its derivatives, and many other drugs were discovered from natural products [5,6,7]. Therefore, phytotherapeutics can be potent and effective products. When they are concomitantly taken with conventional drugs, interactions may take place, with modulation of the clinical effects of the latter. In general, the same mechanisms underlying the conventional drug–drug interactions occur between phytotherapeutics and conventional drugs, generating pharmacokinetic and pharmacodynamic interactions that can be both beneficial and detrimental. When a pharmacokinetic interaction occurs, the phytotherapeutic modifies the absorption, distribution, metabolism, protein binding, or excretion of a drug, thereby altering the levels of the drug or its metabolites. On the other hand, pharmacodynamic interactions can take place when a plant-derived substance produces additive, synergistic, or antagonist activity with respect to the conventional drug [4]. Antagonism is observed when the effects of two compounds are lower than the sum of each one, while quite the reverse is observed in additive interactions given that, in this case, the effects of the two compounds are the sum of each one [2]. For instance, through the co-administration of sedative botanicals such as valerian with benzodiazepines or other hypnotic compounds, a greater hypnotic effect could occur. Conversely, for instance, an antagonistic interaction is observed when a plant with high caffeine content—such as guarana—is taken with a sleep-inducing drug [4]. The other mechanism by which plant derivatives exert their potential when administered with conventional drugs is synergism—a positive interaction that occurs when two compounds are given in combination, resulting in an inhibitory effect greater than the simple sum of the effects of the single drugs administered individually [8,9]. It is worthy of note that the definition of synergy may overlap with that of potentiation, and concrete definitions may derive from a mathematical approach, as demonstrated by the different methodologies developed to evaluate drug–drug interactions, such as in the case of the works of Berenbaum, providing the basis for its use in pharmacology and phytopharmacology [2]. However, this discussion falls beyond the scope of this review and, therefore, we refer to more proper literature reports on the topic [10,11].

**Figure 1:**
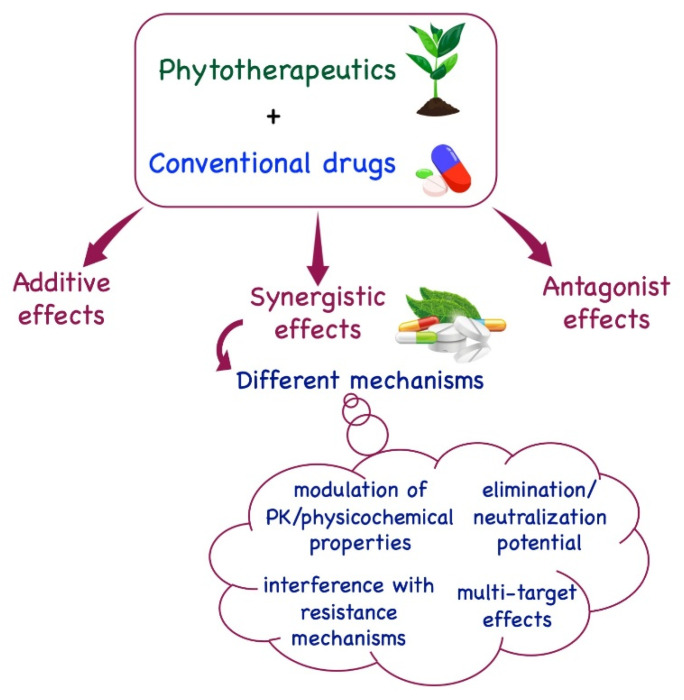
Depiction of the main mechanisms involved in synergism between plant derivatives and conventional drugs.

Several works have been reported in recent decades describing the synergistic effects between plant-derived bioactives and conventional drugs. These effects are generally based on four different mechanisms: the synergistic multitarget effects (when plant derivatives impact on different targets and not only on a single one) [12], the modulation of pharmacokinetic or physicochemical effects (when synergism influences the physicochemical properties, including solubility, of a compound, improving its bioavailability) [13], the interference with resistance mechanisms (when plant derivatives antagonize the onset of drug resistance observed with antibiotics or anticancer drugs against drug-resistant microorganisms or cancer cells) [14], and the elimination or neutralization potential (when a plant derivative is capable of reducing or neutralizing the toxicity of a drug, impacting its adverse effects and improving treatment) [15,16] (Figure 1).

In any case, it is noteworthy that the most common mechanisms described in the literature are synergistic multitarget effects [2]. In recent years, the synergistic effects between phytotherapeutics and conventional drugs have been described in different areas of therapy, based on the mechanisms described above, and many of the commonly employed drugs are based on the synergistic interactions with plant derivatives. For instance, synergistic drug combinations have been envisaged as a promising approach for antimicrobials, with different combinations described as promoters of reducing the minimum inhibitory concentrations of antibiotics for different bacterial strains. Indeed, a promising approach to hinder drug resistance is searching for new and alternative sources of antibacterial molecules that may block the bacterial growth via different mechanisms with respect to the antibiotics that are currently in use, reinforcing the efficacy against drug-resistant microorganisms [17,18,19,20,21,22]. Furthermore, beneficial associations between conventional chemotherapeutic agents and plant derivatives have been described in the field of anticancer research, both as preventive approaches and targeted-oriented strategies [2,23,24], thanks mainly to the synergistic effects lying in the resistance mechanisms, such as the capability of natural bioactives to antagonize drug resistance, enhance drug properties, or mitigate possible side effects, as mentioned above. 

Other examples can be found for pain treatment, as in the case of the association between acetylsalicylic acid, paracetamol, and caffeine [25].

Caffeine is a natural stimulatory compound that is found in several plants, including coffee beans and tea leaves [26]. It is widely known that caffeine has several biological effects and, most recently, a growing body of evidence has highlighted the synergistic effects of this plant-derived compound in association with different conventional drugs in a variety of therapeutic fields. Thus, the primary aim of this review is to provide an overview of the synergistic therapeutic effects of caffeine and conventional drugs, summarizing the progress reported to date. The literary search strategy was implemented using the PubMed, Google Scholar, and ScienceDirect databases, choosing the following keywords: “caffeine”, “synergy”, and “drug (s)”. Works reporting only the co-administration of caffeine with conventional drugs, without deepening the synergistic effects between them, were intentionally excluded.

**Figure 2:**
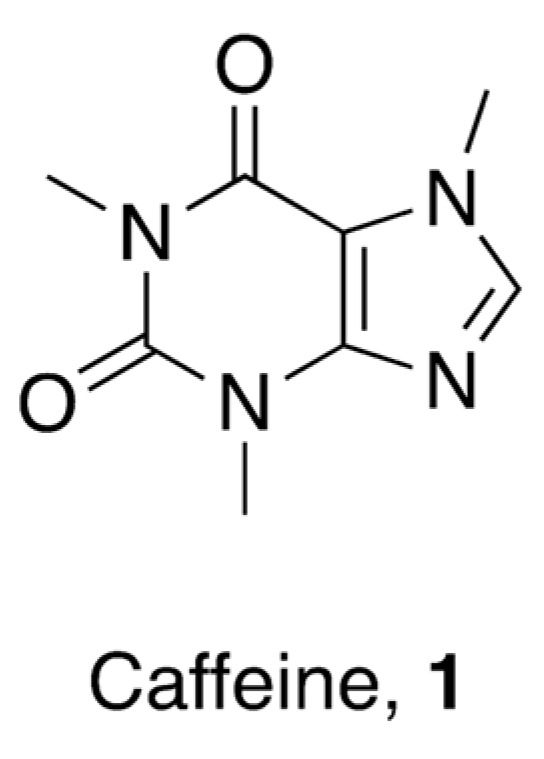
Chemical structure of 1,3,7-trimethylxanthine (caffeine).

Caffeine (1,3,7-trimethylxanthine, **1**, Figure 2) is a pure alkaloid that naturally occurs in the seeds, nuts, and leaves of various plants—mainly in coffee beans, tea leaves, guarana berries, cocoa beans, and kola beans, among others. 

Caffeine is a central nervous system stimulant that is also endowed with various positive therapeutic effects, some of which have not yet been entirely mechanistically clarified. It is the most commonly consumed psychostimulant agent worldwide [27]. Caffeine is structurally related to other natural xanthines (such as theobromine or theophylline) and to some endogenous purine derivatives, such as the nucleobases adenine and guanine or the metabolite uric acid. Caffeine is a drug-like compound given that it complies with the rule of five, it is lipophilic, and it can cross biological membranes [28,29]. 

Caffeine has several pharmacological roles and is widely used as a drug in different therapeutic fields. 

## 2. Biological Activities of Caffeine

### 2.1. Molecular Activities

Caffeine has been widely studied in numerous biochemical assays thanks to its numerous pharmacological roles. The best-described molecular activity of caffeine is the modulation of adenosinergic signaling, being an adenosine receptor antagonist to all subtypes with IC_50_ values in the micromolar range [30]—an activity that most of its biological effects in humans are believed to rely on, as for that of its analgesic adjuvant and neuroprotective properties [26], not to mention its effects in cancer immunotherapy [30]. Among the other numerous activities reported so far, the best characterized and more likely to have important pharmacological consequences are the inhibition of cyclic nucleotide phosphodiesterases (which is among the first activities to be disclosed) [31], the modulation of GABA-A receptors via the benzodiazepine binding site [32], and the inhibition of acetylcholinesterase [33]. Moreover, caffeine is thought to play a role in muscle and neuronal function by activating the ryanodine receptors (intracellular calcium channels that are essential for the mobilization of Ca^2+^) [34]. Several other molecular activities of caffeine have been reported so far; even so, this is beyond the scope of the present review and, therefore, we refer readers to other, more specific reviews for further knowledge (e.g., see Ref. [26]).

### 2.2. Pharmacological Effects 

Caffeine has different and very attractive pharmacological activities, playing several roles as a drug. It is a bronchodilator used for treating asthma, and it is on the WHO list of essential medicines for its respiratory stimulant effect to be used in neonates [35,36]. Indeed, after evidence arising from several clinical trials, it has been used to treat neonatal apnea—especially in cases of prematurity [37]. As for xanthines in general, the main mechanism of action of this therapeutic effect is the antagonism of the adenosine receptors A1 and A2a [38]. 

Furthermore, caffeine is used as an analgesic adjuvant in diverse co-formulations with nonsteroidal anti-inflammatory drugs (NSAIDs)—such as ibuprofen, acetylsalicylic acid, or paracetamol—for treating pain (mainly headache and mild pain), leading to an improvement in the pain relief [39]. Being an analgesic adjuvant, caffeine does not possess an analgesic effect on its own, but it enhances those of other analgesic drugs [40]. A possible explanation can be ascribed to the modifications of the bioavailability and pharmacokinetics of NSAIDs by caffeine, given that it is a substrate and inductor of the cytochrome P450 (CYP) enzyme CYP1A2 [41], interfering with different drugs that are partially or entirely metabolized by CYP1A2 (e.g., antidepressants, antipsychotics, NSAIDs, and cardiovascular drugs). It has also been speculated that the analgesic adjuvant effect of caffeine may be due to its ability to rapidly decrease the gastric pH, thereby increasing the absorption of analgesics and influencing their pharmacokinetics [39]. However, there are reports describing this effect of caffeine in combination with NSAIDs as mediated via A2a antagonism, such as that observed for anti-asthma treatment [42,43]. This A2a-mediated activity involves the vasoconstrictor effect of the A2b blockage [44]. Several clinical trials have shown that co-administration of caffeine with paracetamol or acetylsalicylic acid enables a reduction in the administered dose of the analgesic drug when taken in combination with caffeine to achieve a similar analgesic effect for different types of pain (i.e., postpartum uterine cramping, episiotomy pain, postsurgical pain, or headache) [25]. Moreover, given that caffeine is a brain penetrant, several effects have been observed in the prevention of neurodegenerative diseases, such as Alzheimer’s disease (AD) and Parkinson’s disease (PD). Indeed, several epidemiological studies have shown a significant inverse association of caffeine consumption with AD [45]. Moreover, studies carried out on transgenic mice highlighted an improvement of memory performance, a rehabilitated memory function, and diminished brain amyloid-β levels following 2–4 months of treatment with caffeine [46,47]. Importantly, an inverse association between caffeine administration and the risk of AD has been observed in humans [45]. Furthermore, a correlation between caffeine ingestion and lower PD risk has also been reported. Indeed, different studies have shown that the use of caffeine can reduce the risk of PD, without cardiovascular side effects or incidence of cancer [26]. From a mechanistic point of view, the neuroprotective effects can be ascribed to different mechanisms, such as adenosine receptor antagonism, modulation of cerebral blood flow, and increased oxygen consumption and cerebrospinal fluid production [48]. In particular, the blockage of the A2a receptors was discovered to be capable of preventing amyloid-β-induced toxicity in the brain [49] and tau hyperphosphorylation, leading to an improvement of the disease symptoms [50].

A still-open topic of discussion concerns the effects of caffeine on cardiovascular diseases, given that increases, decreases, or no effects on the cardiovascular risk have been reported. Several factors affect the comparability of the different studies described in the literature (e.g., the different definitions of the amount of caffeine ingested, being sometimes indicated as “cup” or in milligrams; the lack of a placebo; the missed consideration of other parameters influencing the study; the scarce number of participants, affecting the trial’s statistical significance) [26]. Therefore, conclusive considerations are difficult to disclose and, in general, one might state that a moderate intake of caffeine (between 100 and 400 mg per day) may provide some protective effects against the cardiovascular risk, while higher consumptions are more likely to not have such effects. 

In addition to the well-known pharmacological activities of caffeine alone or as an adjuvant with the other drugs described above, several other studies have been reported to date about the use of caffeine in combination with conventional drugs characterized by varied variety of therapeutic indications, spanning from antibiotic activity to anticancer or antidiabetic drugs, among others. Hence, a discussion of the most recent reports describing the synergistic effects of caffeine in combination with conventional drugs is reported here. 

## 3. Caffeine and Antimicrobial Drugs

Antimicrobial resistance constitutes one of the most questionable issues in public health and one of the main causes of death worldwide [51]. Misuse and overuse of antibiotics in clinical practice and in agriculture has resulted in this emergence and led to a remarkable decrease in drugs’ potential to successfully manage infectious diseases [52,53]. Hence, the need to find innovative solutions remains evident. Interestingly, caffeine has shown strong synergistic or additive effects with antibiotics in the case of infections caused by microorganisms belonging to the most worrying human pathogens—with high prevalence of multidrug-resistant isolates, and which increasingly require new therapeutic options. Structural modifications of caffeine could be exploited to increase its adjuvant effects on antibiotic drugs, and its non-systemic use could be directly applied in the clinic in the age of multidrug-resistant pathogens. 

### 3.1. Caffeine and Antibacterial Agents 

**Figure 3:**
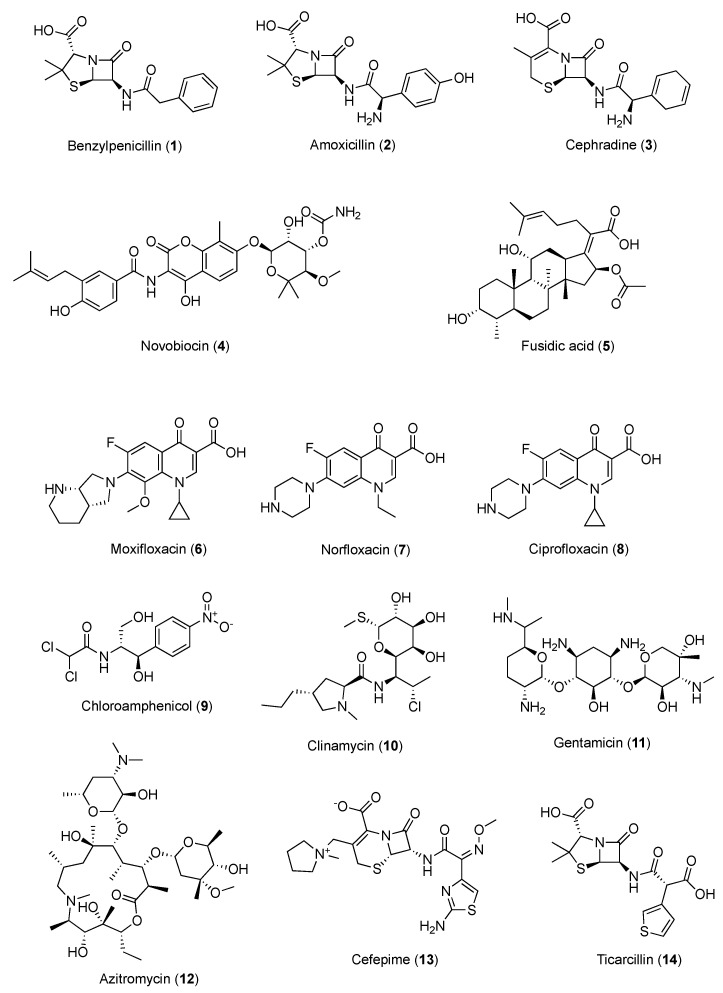
Chemical structures of antibacterial agents showing synergism with caffeine.

The in vitro potential of caffeine to modify the antibacterial properties of well-established antibiotics was examined, representing diverse classes with distinct antimicrobial mechanisms, and various trends could be observed within drug families. Considering *Staphylococcus aureus*, while no significant effect of caffeine was reported against the strain Mu, a slight improvement in drug activity was observed in the *S. aureus* Newman strain when penicillins, carboxypenicillins, and most cephalosporins were used in combination with caffeine, but there was no effect with ampicillins. In particular, the MIC of benzylpenicillin (**1**, Figure 3 vs. *S. aureus* was enhanced by 59 and 40 times with caffeine at concentrations of 5 and 10 mg/mL, respectively, whereas higher concentrations showed no inhibition of the *S. aureus* growth. At the same concentrations, caffeine reduced the MIC of amoxicillin (**2**, Figure 3) vs. *S. aureus* by 22 and 25 times, respectively, whereas that of ampicillin was lowered by 6 and 8 times, respectively [54]. The underlying mechanism of this antimicrobial synergistic interaction is not yet clear. It was hypothesized that the inhibition of bacterial cell walls by penicillins might ease the penetration of caffeine into the bacterial cells, causing a higher concentration of caffeine in the cells and, thus, enhancing the DNA damage [55]. Nonetheless, the synergistic effect may also be due to the inhibition of the *Staphylococcus* penicillinase enzyme, which increases the activity of the penicillinase-sensitive antibiotics. Moreover, the relationship between the polarity of the antibiotics and the effects of caffeine co-administration was also examined. Caffeine seemed to decrease the antibacterial potential of the less polar antibiotics while enhancing that of the more polar ones, probably due to the effect on the physicochemical interaction between caffeine and the drugs in vitro. Conversely, complexation with caffeine through the establishment of van der Waals interactions might reduce the in vitro activity of the less polar antibiotics. Subsequently, against the same *S. aureus* Newman strain, the association of cephradine (**3**) and caffeine resulted in a stronger inhibition, and the activity of cefotaxime and cefepime increased in a moderate manner. Bacitracin, vancomycin, and colistin seemed not to be influenced by caffeine, while aminoglycosides, macrolides, and novobiocin (**4**, Figure 3) exerted a significant potentiation of anti-staphylococcal activity in the presence of caffeine. No modulatory action of caffeine was found with fusidic acid (**5**, Figure 3) and tetracyclines [56]. In general, with regard to other pathogens, caffeine seemed to considerably inhibit the antibacterial activity of fluoroquinolones such as moxifloxacin (**6**), norfloxacin (**7**), and ciprofloxacin (**8**), as well as that of chloramphenicol (**9**) and clindamycin (**10**) (Figure 3) [57,58]. Considering that the antibacterial activity of the fluoroquinolones stemming from the inhibition of bacterial gyrase and topoisomerase IV, the inhibitory effects of caffeine against these drugs can be ascribed to caffeine’s potential to influence the biological activity of molecules that are able to direct covalent [59] or noncovalent [60] interactions with DNA, and which exert their effects through DNA-related enzymes involved in DNA synthesis or maintenance. Similarly, the inhibitory effects of caffeine on aromatic chloramphenicol might be explained by the potential of caffeine to reduce the bioavailability of aromatic compounds, blocking them in noncovalent stacking complexes [61]. When gentamycin (**11**), azithromycin (**12**), cefepime (**13**), novobiocin, and ticarcillin (**14**) (Figure 3) were selected for the evaluation of the possible effects of caffeine association on highly relevant human bacterial pathogens—such as *S. aureus* MSSA ATCC 25923 and MRSA 43300 strains, *P. aeruginosa* ATCC 27853, *A. baumannii* ATCC 19606, and *K. pneumoniae* ATCC 700603—the caffeine enhancement effects were proven to vary according to the pathogens evaluated. Altogether, the strongest increase in the antibacterial activity induced by caffeine was reported vs. the *S. aureus* MRSA strain, *P. aeruginosa*, and *A. baumannii*, while modest effects on the selected antibiotics were observed for *S. aureus* MSSA and *K. pneumoniae*. Among these antibiotics, the effect of caffeine was the greatest for cefepime and azithromycin, while the weakest was for novobiocin. Additionally, synergistic effects of caffeine for at least one bacterium were reported for all antibiotics tested, except for ticarcillin. Overall, low concentrations of caffeine (≥1 mg/mL) considerably diminished the MIC of the preselected antibiotics [56].

### 3.2. Caffeine and Fluconazole 

**Figure 4:**
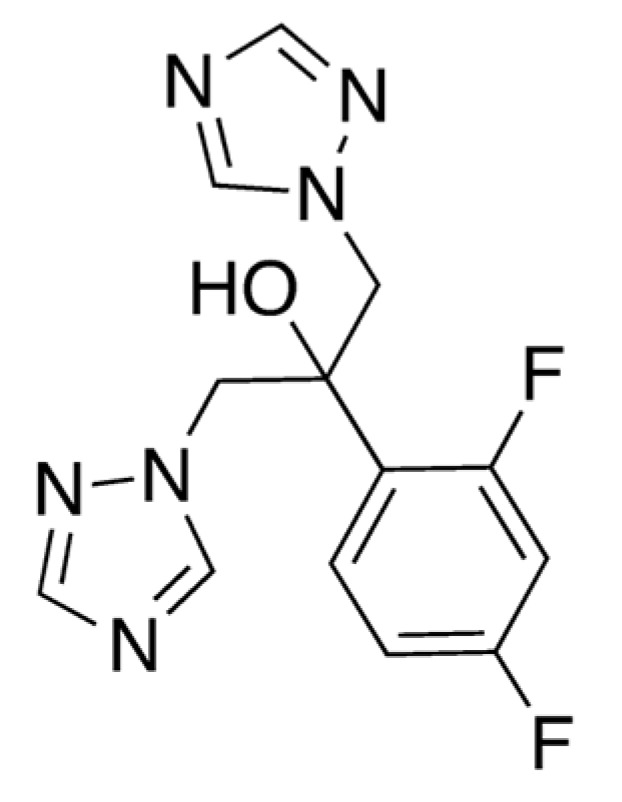
Chemical structure of fluconazole (**15**).

As a component of the human microbiota, *Candida albicans* represents a peculiar opportunistic pathogen that causes candidiasis in individuals who have a latent scarce immune condition, and the widespread emergence of these infections can be serious [62]. Fluconazole (FLU, **15**, Figure 4) is the most frequently utilized azole for both the cure and prevention of candidiasis, acting as fungistatic drug, and is targeted to the crucial enzyme lanosterol 14a-demethylase—also known as Erg11—in the ergosterol biosynthesis pathway [63].

However, the prolonged use of FLU has recently led to the prevalence of FLU-resistant *C. albicans*. Alternative treatments and solutions are being evaluated, and in this case caffeine could also represent a strategic ally to curb this issue. Okubo showed that 2.5% black tea extract inhibited the growth of filamentous fungi [64], and at more than 10% concentration it inhibited the growth of *C. albicans*. In more detail, the combination of caffeine and FLU was evaluated for the potentiation of antimitotic activity against *C. albicans* PTCC-5027. Concentrations of 10 and 50 mg/L FLU in the presence of 6.25 mg/L caffeine resulted in 91.8% and 98.9% inhibition of the strain’s growth, respectively. A stronger growth inhibition was obtained with a twofold increased quantity of caffeine. Considering the absence of any inhibitory effect of less than 50 mg/L FLU on the PTCC5027 strain, the effect of the combined use of 10 and 50 mg/L of FLU and 12.5 mg/L of caffeine was discovered to be 99.3% and 99.7%, respectively, compared with FLU and caffeine alone. The association of caffeine and FLU was tested in an attempt to reduce the effective dose of FLU by using a FLU concentration lower than the MIC, and the resulting combination was proven to be fungicidal rather than fungistatic. The mechanism of the synergistic effect remains unknown but could involve cytoplasmic membrane damage to fungi [65].

## 4. Caffeine and Anticancer Agents

**Figure 5:**
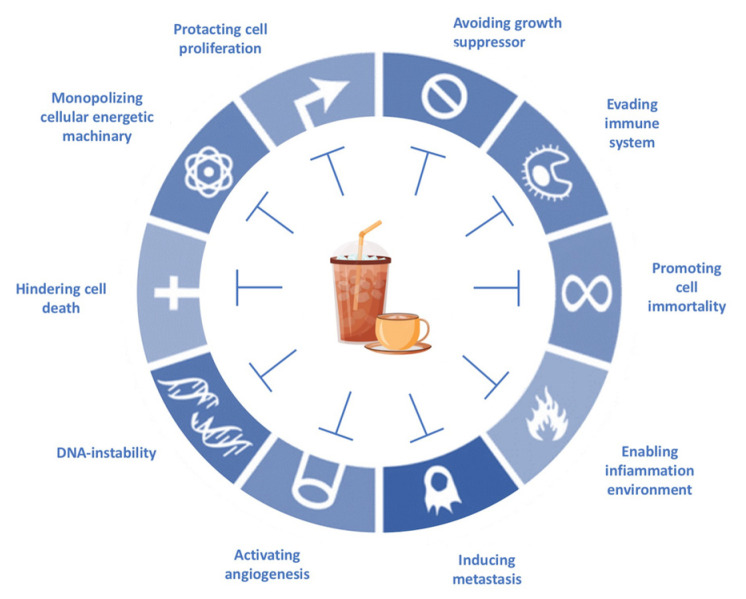
Coffee components target the hallmarks of cancer, according to Hanahan and Weinberg’s hallmarks of cancer (2011), with modifications.

Epidemiological evidence proves that caffeine consumption exhibits anticancer properties. Caffeine-based beverages offer a natural source of compounds that target the hallmarks of cancer, and caffeine and related xanthine derivatives have also been applied in experimental chemotherapy thanks to the idea that some molecular pathways—such as apoptosis and DNA damage repair pathways—could be influenced by these alkaloids [66,67] (Figure 5).

Moreover, it has recently been clarified that the effects of some antiblastic drugs are also enhanced by combination with caffeine. Even though it has been suggested that the mechanism of caffeine synergism could differ from one drug to another, it seems that this natural compound enhances the efficacy of anticancer agents by affecting drug-induced cell-cycle arrest. There have been various reports that caffeine seems to show an inhibitory effect on DNA repair, damaging the genome. Whether caffeine possesses anticancer properties remains as a question to be clarified. However, caffeine intensifies successive cell apoptosis, overcomes radiotherapy- or chemotherapy-induced delays in cell-cycle progression, and enhances anticancer agents and radiation toxicity. Nakata et al. [68] tested caffeine with ifosfamide (**16**) using an innovative drug delivery system with Span 80 nanovesicles, and the results demonstrated clear antitumor effects, without exerting adverse effects. Caffeine enhanced the anticancer properties of ifosfamide in different tumor cell lines by interfering with DNA damage repair processes and potentiating the induction of apoptosis. Although the anticancer drug armaments continue to grow, acquiring new entities such as the most modern biologics, alternative treatments are being evaluated to solve this issue. Caffeine represents a widely investigated natural molecule thanks to its global diffusion and uses, and it has been found so far to be a valid ally to enhance anticancer drugs’ effects or bypass acquired drug resistance. Various well-known antitumor drugs are being studied in combination with caffeine, and interesting data could provide an interesting perspective on the consumption of this alkaloid (Table 1). In specimens, alkylating agents and DNA-targeting drugs are the most common drugs studied in combination with caffeine, and these drugs exert the most pronounced synergistic effects via this combination. Among them, doxorubicin, cisplatin, cyclophosphamide, mitomycin C, and methotrexate are the most noteworthy.

When administered with doxorubicin (**17**), caffeine and its analogue theobromine exhibit inhibitory effects on the doxorubicin efflux from cancer cells, enhancing the drug’s antitumor effects [69]. Motegi et al. [70] affirmed that xanthine derivatives, caffeine, and theophylline generated synergistic antitumor effects in a canine hemangiosarcoma cell line in combination with or without doxorubicin. The antitumor properties were assessed by counting the cancer cells’ growth, apoptosis, and cytotoxic effects on cells, measuring the caspase-3/7 or annexin V protein gene expression. The results showed that caffeine and theophylline induced apoptosis in those cancer cell lines. In contrast to the previous study, Mohammadrezaei et al. demonstrated rather different findings [71]. By analyzing the induction of senescence in MCF-7 breast cancer cells induced by doxorubicin via ATM and chk2 activation, the authors stated that the sensitivity of the cells to this induced senescence could not be potentiated by a combination of a chk2 inhibitor, caffeine, and doxorubicin, nor by caffeine or the chk2 inhibitor alone. Tsuchiya et al. [72] described how 36 sarcoma patients with histologically high-grade soft tissue were treated with doxorubicin, cisplatin, conservative surgery, and caffeine-potentiated chemotherapy. Among them, nine patients had stage III disease, eight of whom died of metastatic disease within two and a half years from the beginning of the treatment. The authors affirmed that limb-sparing surgery and caffeine-enhanced chemotherapy resulted in beneficial effects for stage II non-metastatic soft-tissue sarcomas, while the stage III metastatic soft-tissue sarcomas remained unresolved. 

Cisplatin (CDDP, **18**), the most common platinum complex widely used for treating solid tumors, shows the most synergistic effects when associated with caffeine. CDDP toxicity and chromosome breakage are increased by the alkaloid. CDDP’s cytotoxic effects result in the formation of DNA interstrand crosslinks (ICLs)—highly toxic DNA lesions that prevent strand separation. When patients were treated with 4 mg of caffeine once per day for three days after the administration of CDDP, a pronounced sarcoma regression was observed in cohorts treated with 5 or 10 mg/kg CDDP, without significant weight loss. Moreover, caffeine increased the antitumor effect of CDDP on transplanted osteosarcoma in BALB/c mice [73]. Karita et al. [74], utilizing polyethylene glycol liposomes containing cisplatin (CDDP-L) as a new delivery system, found that the antitumor effect of CDDP-L in osteosarcoma-bearing rats was boosted not only by the co-administration of caffeine, but also by the increasing dose of the alkaloid. In hepatocellular carcinoma (HCC), it was confirmed that caffeine plays a crucial role in inhibiting the DNA repair process. The homologous recombination (HR) DNA repair is stimulated by ICLs via the Fanconi anemia (FA) pathway. FA is a disease in which imperfect DNA repair induces bone-marrow failure and the development of solid tumors, especially in the liver. ICL-inducing agents sensitize FA cells, and it is clear that FA pathway inhibition enhances the anticancer effect of CDDP. Moreover, recently, it seems that the FA pathway is also implicated in repairing CDDP-induced DNA damage. In this scenario, caffeine boosts the anticancer effect of CDDP in HCC cell lines via inhibiting DNA repair by suppressing HR [75]. Oda et al. [76] suggested that this effect could be ascribed to the interaction with the FA pathway, despite no suggestions having yet been offered for a relationship between the FA pathway and caffeine, and the mechanisms responsible for this synergism require further clarification. In the same study, it was highlighted that associated application of caffeine (200 µg/mL) significantly decreased the IC_50_ value for CDDP. While caffeine alone has no effect on cell proliferation, in combination with CDDP, a caffeine concentration of ≥100 µg/mL causes a reduction in cell viability. The same trend was observed when studying apoptosis: caffeine alone had no effect on HepG2 cell apoptosis; on the other hand, if cisplatin treatment enhanced the level of apoptosis (14.5 ± 2.9%) compared with the untreated control group (2.6 ± 0.1%), the level of apoptosis was enhanced by one and a half times by the association between CDDP and caffeine (24.0 ± 0.9%). It was also observed that caffeine-induced cell-cycle arrest arose at the G1 phase and decreased the proportion of cells in the S phase [77]. Compared with the relevant controls, CDDP treatment alone provokes a reduction in the proportion of viable cells in the G1 phase but enhanced the number of S-phase cells. Notably, caffeine inhibited this CDDP-dependent increase in the proportion of viable cells in the S phase. Cells with damaged DNA show a prolonged G2 phase to accommodate increased DNA repair. Caffeine is believed to shorten the G2 phase by accelerating DNA synthesis, and this results in the cell entering the mitotic phase without adequate DNA repair. Abe et al. analyzed the combination of caffeine and CDDP in the treatment of osteosarcoma. 

Osteosarcoma is an uncontrollable primary bone tumor that mainly develops in young adults and adolescents, and its 5-year survival is approximately 70%. First-line therapy is based on high-dose methotrexate, CDDP, doxorubicin, and ifosfamide, despite these regimens having not improved outcomes. New kinase inhibitor drugs such as pazopanib, trabectedin, and eribulin have been introduced recently, but they are not as effective as the first-line chemotherapy. Studying the effects of cisplatin combined with the other drugs on tumor cell survival, it was demonstrated that cisplatin, cisplatin + caffeine, cisplatin + citrate, and cisplatin + caffeine citrate inhibited the viability of all cells in a dose-dependent manner. IC_50_ values were registered after 72 h of treatments with cisplatin + caffeine citrate for LM8, HT1080, and HOS cell lines, and they were 0.30 μmol/L, 1.63 μmol/L, and 1.16 μmol/L, respectively. Caffeine overcomes cisplatin-induced S/G2 cell-cycle arrest, with subsequent increased apoptosis in HOS cells. Cell-cycle arrest is a survival mechanism in chemotherapy-treated cells, and caffeine-induced progression of the cell cycle resensitizes cancer cells to chemotherapy. Moreover, it was demonstrated that caffeine induced G0/G1 cell-cycle arrest and suppressed cell proliferation. Caffeine citrate had stronger effects as a combination drug than caffeine and citric acid in both defeating the cisplatin-induced S/G2 cell-cycle arrest (with subsequent increased apoptosis) and provoking G0/G1 cell-cycle arrest (with subsequent suppressed cell proliferation) [78]. 

Igarashi et al. [79] evaluated the efficacy of caffeine and valproic acid (**19)**—in combination and alone—against osteosarcoma. While monotherapy was dose-dependently active against osteosarcoma cells, the combination therapy had greater efficacy against all cell lines and was synergistic for the 143B and MG63 cell lines at a caffeine concentration of 1 mM. Both drugs induced higher activity at lower concentrations in combination, along with high caspase activity. Each agent induced apoptosis in a time- and dose-dependent manner. In more detail, combination therapy provoked apoptosis at lower concentrations compared to monotherapy. In an orthotopic mouse model, while caffeine and **19** monotherapies did not inhibit cancer cell growth, the combination led to a more pronounced decrease in cancer volume.

Moreover, a different class of drugs (antimitotic drugs) was also studied in combination with caffeine. Gururajanna et al. [80] illustrated that caffeine and Taxol (**20**) provoke arrest, inhibit cell growth, and eventually lead to the death of pancreatic adenocarcinoma cells. Their study demonstrated that these effects were reduced in p53-mutant cells and increased in wild-type p53 cells, suggesting an essential role of p53 in establishing the effects of these agents in pancreatic cancer cells.

An interesting study by Popovic et al. [81] was conceived to investigate the combination of caffeine and metformin (**21**). Despite not being an anticancer drug, metformin exhibits various antitumor effects, which could be potentiated by the combination with other drugs or natural compounds. Metformin has been one of the most revolutionary antidiabetic drugs of the past century thanks to its ability to decrease glycolytic capacity. Moreover, this drug is able to decrease mitochondrial respiration and energetic efficacy in lymphocytic leukemia cells in vitro. It also causes ATP depletion, accumulation of AMP, and phosphorylation of AMP-activated protein kinase (AMPK), which is a downregulator of the Warburg effect. All of these aspects result in metformin inhibiting tumor progression. Recently, it has been demonstrated that **21** inhibits matrix metalloproteinase-9 (MMP-9) activity, as well as the invasion and migration of human fibrosarcoma cells in vitro, via Ca-dependent signaling pathways. Therefore, metformin has the potential to be an anti-sarcoma drug [82,83]. Metformin can also act as an antifolate, which secondarily induces the ATM kinase and downstream AMPK in breast cancer cells in vitro by inhibiting DNA replication and cell proliferation. Hence, appropriate combinations may improve metformin’s efficacy. The synergistic anticancer action of metformin and the natural antioxidant caffeic acid was evident through the regulation of mitochondrial metabolism in cervical carcinoma cells. Peroral treatment with a metformin–caffeine combination in fibrosarcoma-induced hamsters significantly inhibited tumor growth. Histopathological and immunohistochemical assays revealed a decrease in tissue penetration, an expansion of necrosis and hemorrhagic areas, and a reduction in vasculature in all analyzed slices of tumors treated with this combination when compared with controls. The treatments had no significant effects on the body weight of the animals during the study.

The antitumor activity of 5-fluorouracil (5-FU, **22**) and caffeine in combination was also examined to curb hepatocellular carcinoma (HCC). Wang et al. analyzed the effects both in vitro and in vivo. In comparison to 5-FU or caffeine monotherapy, the combination of 5-FU and caffeine inhibited the proliferation and provoked the apoptosis of cancer cells by increasing the generation and accumulation of ROS. No pronounced difference in antiproliferative effects was found between 25 and 50 μM 5-FU combined with 1 mM caffeine, both of which had synergistic effects, suggesting that caffeine can sensitize HCC cells to lower doses of 5-FU [84].

## 5. Caffeine and Anti-Inflammatory Drugs

Caffeine has been widely studied and reported for its anti-inflammatory properties, causing an increase in the analgesic response to NSAIDs, but only in particular pain states and at certain dose ratios. These findings have led to the use of caffeine as an analgesic adjuvant in formulations based on combinations with a variety of nonsteroidal anti-inflammatory drugs (NSAIDs). Several hypotheses have been formulated about the mechanisms by which caffeine can influence NSAIDs’ analgesic effects. A variety of evidence suggests the implication of both pharmacokinetic and pharmacodynamic factors in this potentiation effect. In particular, it has been proposed that caffeine is able to augment the absorption or to decrease the elimination of NSAIDs, thereby increasing their bioavailability. However, in vivo studies did not support this hypothesis. Other hypotheses suggested that NSAIDs could be responsible for the increase in caffeine’s bioavailability, since caffeine itself possesses an intrinsic anti-nociceptive effect. Nonetheless, experimental studies revealed that caffeine’s bioavailability was not remarkably modulated by the concomitant administration of NSAIDs. Given the fact that pharmacokinetic interactions have not been proven, a pharmacodynamic mechanism has been put forward to justify the improvement of the anti-nociceptive response. However, even in this case, the mechanism of the anti-nociceptive adjuvant effect of caffeine has not been completely clarified to date [85]. 

**Figure 6:**
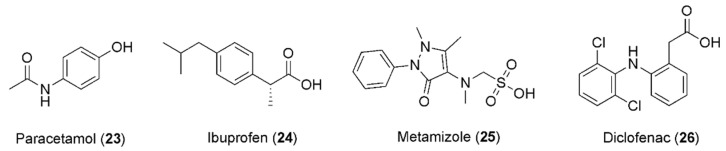
Chemical structures of anti-inflammatory drugs showing synergism with caffeine.

In the past few years, the combination of caffeine and paracetamol (**23**, Figure 6) has been largely explored, leading to commercialization of this combination. In particular, in 1993, Granatos-Soto et al. published the outcomes of a study on a pain-induced functional impairment model in rats, considering the potential synergistic effect of caffeine and paracetamol. Combinations of 316 mg kg^−1^ paracetamol with different caffeine doses led to analgesic effects remarkably greater than that of paracetamol alone. Further experiments suggested a mechanism involving pharmacodynamic factors [86].

In this context, it is well known that caffeine does not alter the plasma level of ibuprofen (**24**, Figure 6) and that ibuprofen does not affect the bioavailability of caffeine [85], raising the possibility that the anti-nociceptive effect caused by caffeine is observable as a result of the impediment of the peripheral pro-nociceptive effect of adenosine. In fact, a better mood can be observed because the central nervous system and supraspinal noradrenergic pathways are activated [87]. Notably, it has been reported that the dose of ibuprofen can be reduced in response to the addition of caffeine, while the healing effect of the drug remains the same. In most studies, ibuprofen was utilized with 100–130 mg of caffeine, in patients experiencing headaches, postoperative dental pain, and postpartum pain. In about 20–25% of patients, the pain was attenuated after co-administration of NSAIDs and caffeine, in comparison to 10–15% treated with NSAIDs only. The helpful impact of caffeine on the analgesic effects of ibuprofen and other NSAIDs has been proven in the management of headaches in children. The best advantageous effect was detected after third-molar removal, with 200 mg of ibuprofen supplemented with 100 mg of caffeine [88].

Diaz Reval et al. examined the effect of the addition of caffeine on the enhancement of the anti-nociceptive effect of metamizole (**25**, Figure 6) in a formalin model, showing that metamizole produced a dose-dependent anti-nociceptive effect with SD_50_ = 329.61 mg/kg. Interestingly, caffeine also showed anti-nociceptive effects at 3.16, 10.0, 17.8, and 31.6 mg/kg concentrations. When a sub-effective dose of metamizole (100 mg/kg) was combined with caffeine (3.16, 10.0, 17.8, or 31.6 mg/kg), stronger anti-nociceptive effects were produced than the corresponding effects produced by metamizole alone, highlighting caffeine’s ability to change the effect of metamizole in the inflammatory pain model, in which it also presented an anti-nociceptive effect [89].

A study published in 2013 was conducted to investigate whether the administration of diclofenac combined with caffeine produced anti-nociceptive synergism, using a formalin model to estimate the anti-nociceptive effects produced by the oral administration of diclofenac (**26**, Figure 6), caffeine, or their combination.

Under these experimental conditions, minimal doses of caffeine, which did not display any anti-nociceptive effects, were administered, even when only a 2% formalin concentration was utilized, based on previously reported evidence that the ED_50_ for caffeine is 5 mg/kg when 2% formalin is administered to generate nociception [90]. The results revealed that the caffeine doses were lower than 5 mg/kg and the effects were lower than 50%. These data are consistent with the published reports. When the combination of both drugs was administered, it was clearly noted that caffeine was effective in producing an augmentation of diclofenac’s effect, especially in the first doses. Remarkably, the minimum doses of the two drugs taken together had an increased effect, while when administered separately they had no effect, resulting in an ED_50_ of 6.715 mg/kg for diclofenac [91].

Co-administration of caffeine and glucocorticoids is a habitual therapeutic practice in the neonatal period, as they are both viable instruments to reduce the rates of different antenatal and postnatal complications in preterm infants, such as bronchopulmonary dysplasia (BPD) and respiratory distress syndrome (RDS) [92]. In this regard, Fehrholz et al. examined how the expression of surfactant protein (SP)-B—which is decisive for the physiological function of pulmonary surfactants—is modulated by caffeine and different glucocorticoids. The experiments outlined a synergistic upregulation of SP-B mRNA in H441 and A549 cells, induced by the concomitant use of glucocorticoids and caffeine. This kind of effect was correlated with heightened levels of the glucocorticoid receptor (GR) and of the SP-B transcription cofactor ErbB4. These results indicate that basic SP-B mRNA transcription initiated by glucocorticoids can be enhanced via caffeine-mediated inhibition of PDEs, which provokes an increase in cellular cyclic adenosine monophosphate (cAMP). These findings suggest that the administration of caffeine in combination with glucocorticoids may be beneficial in surfactant homeostasis during the treatment of preterm infants [93,94].

## 6. Caffeine and Opioids

**Figure 7:**
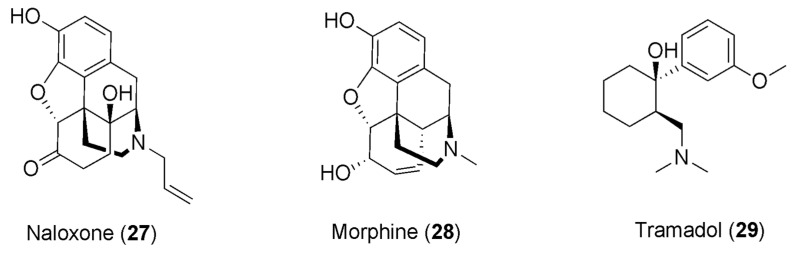
Chemical structures of opioids showing synergism with caffeine.

As stated in the previous paragraph, caffeine has been reported for its capability of enlarging the pain-relieving response to NSAIDs, giving rise to the application of caffeine as an analgesic adjuvant in combination with NSAIDs. Furthermore, it has been observed that by co-administering the opioid antagonist naloxone (**27**, Figure 7) with a combination of the NSAID diclofenac and caffeine, an 84% decrease in the anti-nociceptive effect is accomplished. This could denote a great contribution of opioids in the anti-nociceptive synergistic effect of caffeine with the NSAIDs [91].

The reduction in the anti-nociceptive effect following the administration of naloxone was also detected in rats when treated with caffeine in association with morphine, reversing the anti-nociceptive effect of morphine observed in rats that were treated with caffeine but not exposed to naloxone. The outcomes of this study corroborate the theory of the participation of opioid receptors in caffeine’s adjuvant effect [95].

This evidence induced researchers to analyze the synergistic effects of caffeine in combination with opioid drugs, and several studies have recently reported the adjuvant effects of caffeine in chronic pain patients treated with opioids, even though the mechanism of this potentiation is still intricate and unclear.

In this context, Scott et al. reported a study estimating the consequences of caffeine consumption on pain and other symptoms in opioid-using and non-using chronic pain patients meeting the survey criteria for fibromyalgia. The results of this study showed that patients taking both opioids and caffeine had less severe symptoms compared to those using opioids but not caffeine, with a dose-dependent effect. Moreover, patients not taking opioids but only caffeine showed no differences in pain relief with respect to those not consuming caffeine, except for an improvement in physical function. This suggests that the anti-nociceptive effect of caffeine is achieved only with concomitant use of opioids [96].

The administration of caffeine as an adjuvant in cancer pain treatment has also provoked interest, and some reports have outlined the use of caffeine with opioids in this context. Mercadante and colleagues studied the effects of caffeine infusion in cancer patients treated with morphine (**28**, Figure 7), showing a reduction in pain intensity, albeit a weak one [97]. Another study found more significant pain reduction and drowsiness following caffeine infusion in cancer patients on opioid treatment, even though this effect was clinically relevant to a lesser extent with respect to patients not consuming caffeine [98].

Interestingly, Misra and coworkers found no difference in the ratio between the concentrations of morphine metabolites and morphine itself in the liver when comparing rats treated with caffeine with untreated rats, revealing that caffeine does not condition the metabolism of morphine [95]. Moreover, higher levels of morphine in both the brain and plasma were detected, suggesting the implication of pharmacodynamic components in the synergistic effect of caffeine and morphine. On the other hand, for caffeine and some analgesic drugs, it was also hypothesized that pharmacodynamic mechanisms are implicated in the synergistic effect, as a consequence of their plasma level analyses that ruled out the involvement of pharmacokinetic mechanisms [99,100,101].

Caffeine’s synergism with opioids could also take place thanks to the ability of caffeine to stimulate the release of β-endorphin in the blood [102] and to augment central noradrenaline turnover [103,104]. Indeed, these data suggest that the resulting enhancement in the release of endogenous opioids and the participation of the noradrenergic and adrenergic systems could be the basis for the increase in opioid analgesia exerted by caffeine.

Another study investigated the synergistic interaction of the synthetic opioid analgesic drug tramadol (**29**, Figure 7) and caffeine, using the formalin test and exploring the nociceptive behavior based on the flinching response of the formalin-treated paw. In this study, rats were divided into five groups and received tramadol or caffeine alone, or combinations of tramadol and caffeine. In this test, the opioids usually showed their effects in two phases [105], and the results obtained by the authors were consistent with the literature, with tramadol manifesting a dose-dependent anti-nociceptive effect in both phases. In contrast, caffeine exhibited a dose-dependent anti-nociceptive effect only in the second phase of the test, generating a synergistic effect with tramadol only in phase two. Indeed, a combination of tramadol with caffeine provoked an augmentation in the anti-nociceptive effect with respect to each of them alone. Therefore, the results of this study revealed the ability of caffeine to potentiate the anti-nociceptive effect of tramadol, being capable of causing synergism with this opioid drug, which is broadly used in moderate–severe pain management. Even though the mechanism has not been explicated, it was reported that caffeine is capable of turning on the noradrenergic and serotoninergic systems in anti-nociception estimated in a formalin model [106]. Thus, it was hypothesized that the synergism of the co-administration of tramadol with caffeine could result from the interplay of these molecules with the serotoninergic and opioid pathways of the endogenous analgesic pathway. Moreover, to deepen the synergism analysis, the dose reduction index (indicating the number of times that the dose of each drug is reduced in a synergistic combination) was established, showing that the doses of tramadol in association with caffeine can be lowered by factors of 1–4. This analysis provides a sense of the extent to which the dose can be reduced while retaining its efficacy but reducing the side effects of the opioid, which is of primary importance—particularly in long-term treatments [107].

## 7. Synergistic Effects of Caffeine in the Treatment of Some Diseases

### 7.1. Caffeine and Parkinson’s Disease 

**Figure 8:**
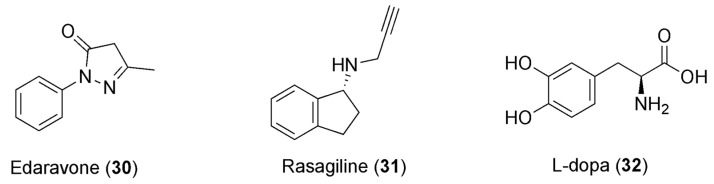
Chemical structures of anti-PD drugs showing synergism with caffeine.

Caffeine consumption elicits beneficiary psychostimulants that have also been associated with a reduced risk of developing Parkinson’s disease (PD) [108]. PD is one of the most frequent neurodegenerative diseases, for which there is currently no cure. It affects more than 1% of the elderly population. Notably, the manipulation of adenosine neurotransmission is recognized as one of the most interesting therapeutic approaches to this disease [109]. This alkaloid was studied in combination with edaravone (**30**, Figure 8) in a chronic rotenone-induced rat model of PD. Rotenone modifies the mitochondrial electron transport chain leading to the generation of ROS, which represents one of the major causes of PD, and its chronic administration is broadly utilized to produce laboratory models of PD. Edaravone is a potent free-radical scavenger, and a three-week treatment with edaravone in combination with caffeine can decrease rotenone-induced oxidative cell stress, muscle weakness, and cognitive impairment. The effect was more notable compared to the group solely treated with edaravone, showing a marked synergistic effect. This could be explained by edaravone’s scavenging of the peroxynitrite free radical. Increased levels of the protein nitroxidation marker 3-NT (3-nitrotyrosine) were detected in the group of animals that were only exposed to rotenone, whereas the concentrations of 3-NT in the control and association-treated group were similar. The combination also re-established memory and muscle strength by combating the effects of the rotenone. Hence, this association could be used for the management of PD in humans—alone or in combination with the currently available pharmacotherapeutic options—after significant clinical data become available [110]. Furthermore, caffeine was also evaluated in combination with rasagiline (**31**, Figure 8) and L-dopa (**32**, Figure 8), for the purpose of verifying the synergistic neuroprotective effects of co-administration against paraquat (PQ)-induced PD in mice.

The co-administration of coffee and caffeine with L-dopa and rasagiline resulted in synergistic effects affecting the dopaminergic system. This association provokes the rebalancing of parameters of neurotransmitters and antioxidant and behavioral responses in murine brain models, along with a defensive effect from histopathological modifications and astrogliosis. A combination of rasagiline and coffee augmented the locomotor activity in a rotarod test with respect to the combination of L-dopa and coffee. Moreover, L-dopa-based combinations reduced the immobility time in the forced swim test compared to rasagiline-based combinations, while concurrent co-administration of L-dopa and caffeine produced a similar increase in locomotor activity compared to co-administration of rasagiline and caffeine. The co-administration of rasagiline and coffee or caffeine also resulted in increased dopamine and superoxide dismutase levels, and in decreased brain levels of malondialdehyde, compared to the respective combination with L-dopa. Conversely, the combination of L-dopa with coffee or caffeine enhances the brain levels of serotonin and decreases those of NO with respect to the rasagiline combination. In general, the neuroprotective effects of caffeine were described in various in vivo PD models via different mechanisms. In particular, the antagonism of the A2A receptor and the induction of the PI3K/Akt pathways’ signaling are the most examined mechanisms [111]. The inhibition of the A2A receptor by caffeine binding means the downregulation of the presynaptic release of glutamate through reducing Ca^2+^ influx, averting immoderate related neurotoxicity [112]. Moreover, the consequent inhibition of microglia by caffeine’s blockage of the A2A receptor provokes the inhibition of the release of inflammatory cytokines, protection from neuroinflammation, and regulation of reactive oxygen species and other factors responsible for neuronal loss [113].

### 7.2. Caffeine and Obesity

**Figure 9:**
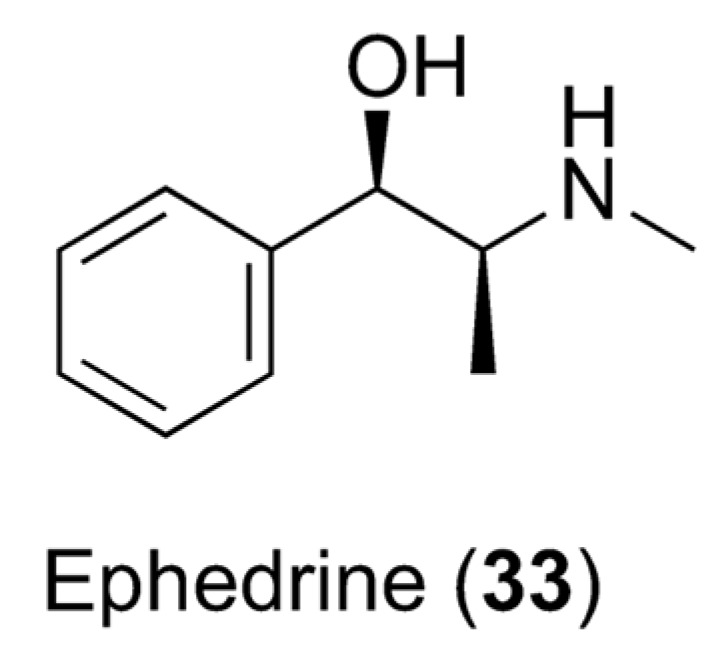
Chemical structure of ephedrine (**33**).

Overweight and obesity are described as anomalous or excessive fat accumulation that constitutes a risk to health. The treatment of obesity is in its infancy, even though recent research has concentrated on finding drugs that can augment lipolysis and the metabolic rate [114]. Previous studies on adipocytes revealed that the combination of catecholamines and caffeine exhibits synergy and results in an increased cAMP rate. Moreover, the combination of 20 mg of ephedrine and 200 mg of caffeine showed very encouraging results: subjects treated with this combination exhibited more pronounced loss of body fat compared to those treated with a placebo [115]. However, ephedra-containing supplements cannot be sold due to concerns over the cardiovascular risks connected with its use, and this association could represent a risk factor. Caffeine is a lipolysis stimulator and a metabolic rate accelerator, as well as amplifying intracellular cyclic adenosine monophosphate (cAMP) levels by inhibiting phosphodiesterase. High cAMP levels are responsible for increased triacylglyceride breakdown and circulating free fatty acids. Regardless, despite caffeine increasing the resting energy expenditure and fatty acid turnover, most of the fatty acids are re-esterified, and weight loss cannot be achieved. On the other hand, the alpha- and beta-adrenergic receptor agonist ephedrine (**33**, Figure 9) was found to augment thermogenesis and metabolism in asthmatic patients. Ephedrine amplifies the release of norepinephrine, which provokes lipolysis by binding to adipocytes. Recently, it has been shown that an association of ephedrine and caffeine could result in a reduction in body fat and be used in the treatment of obesity, but similar conclusions could not be drawn for other related drugs, such as albuterol. The response trends were conflicting, and dissimilarities in the mechanism of action between ephedrine and albuterol may have also contributed to the absence of synergy. Because ephedrine agonizes both α- and β-adrenergic receptors and albuterol remains a selective β2-adrenergic receptor agonist, synergy between ephedrine and caffeine in energy expenditure may be due to additional stimulation of α1-adrenergic receptors [116].

### 7.3. Caffeine and Pioglitazone

**Figure 10:**
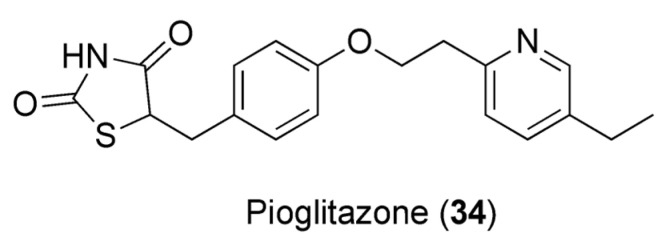
Chemical structure of pioglitazone (**34**).

Pioglitazone (PIO, **34**, Figure 10) is a thiazolidinedione-based drug and one of the antidiabetic medications used in combination with other drugs to curb diabetes mellitus. In order to evaluate the effect of caffeine on the pharmacodynamics and pharmacokinetics of PIO, Alshabi et al. studied the effects of these combinations in rat models. The co-administration of PIO and caffeine elevated PIO’s AUC by 38.5%, its Cmax by 36.5%, and its half-time and tmax. Because of the acidic nature of PIO (pka of 5.19) [117], its bioavailability could be controlled by pH changes. Caffeine decreases gastric pH, preventing drugs’ ionization and increasing gastric and ileac blood flow, with consequent improvement of PIO’s bioavailability. Caffeine also provokes an increased PIO Cmax. This could be attributed to the caffeine-induced decrease in clearance of PIO and increase in gastric emptying time. The reduction in hepatic blood flow, portal blood pressure, and portosystemic shunting, along with the increased mesenteric arterial supply, could also furnish alternative explanations to this result of caffeine vs. PIO. Furthermore, caffeine-derived metabolites may impede the transport and secretion of PIO. Pharmacodynamic data, on the other hand, corroborate the theory that caffeine potentiates PIO’s activity. If PIO alone decreases the blood glucose level by 64.7% after 7 days of treatment, the combination with caffeine results in a synergistic effect, with a reduction of 67.1% after 7 days of treatment [118].

## 8. Conclusions

One way in which plant-derived compounds show their pharmacological capacity is synergism—a positive interplay between two or more compounds, leading to a more pronounced effect than the sum of their individual effects. Many currently used drugs are based on synergistic interactions with phytochemicals, spanning diverse areas of therapy. Among them, caffeine has been demonstrated to be particularly auspicious as a useful synergistic compound with different conventional drugs of various therapeutic classes. Indeed, in addition to its pharmacological activities as a bioactive compound per se, several studies have been recently reported describing its synergistic effects when co-administered with some conventional drugs. In particular, they have delineated the synergistic role of caffeine with antimicrobials—both antibacterial and antimycotic—showing it to stimulate a reduction in the minimum inhibitory concentrations of antibiotics for different bacteria and to enhance the antifungal activity of the antifungal drug fluconazole.

Furthermore, in complex diseases such as cancer, positive synergistic caffeine–drug interactions have been detected, paving the way for the achievement of better outcomes, including identifying appreciable benefits to patients or reducing side effects.

Caffeine has also been extensively reported for its anti-inflammatory properties, consisting in an increase in the analgesic response to NSAIDs, leading to the use of caffeine as an analgesic adjuvant in formulations containing combinations with a variety of NSAIDs. Interestingly, caffeine showed synergistic effects in combination with opioids, with several reports describing the adjuvant effect of caffeine in chronic pain patients treated with opioids, although the mechanism of this potentiation remains complex and unclear.

Caffeine consumption also elicits beneficiary synergistic effects with drugs that are active against Parkinson’s disease, as well as showing positive synergistic effects in some metabolic diseases—such as with ephedrine in the treatment of obesity and with pioglitazone in antidiabetic therapy. Even though the mechanisms underlying the synergism of caffeine with some conventional drugs are not yet fully understood, these recent reports give us an idea of the undoubtedly increasing interest in this field of research and the promising role of caffeine in many therapeutic areas. Furthermore, by taking advantage of the positive synergistic effects of caffeine, we might envision the useful role of this plant-derived compound as support in various therapies, while also providing a means to avoid the harmful side effects of conventional drugs, especially in chronic treatments.

## Figures and Tables

**Table 1:** Cited drugs and their antitumor effects in combination with caffeine.

Drug	Structure	Activity	Caffeine Synergistic Effects
Ifosfamide (**16**)	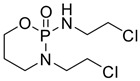	DNA crosslinks by alkylation at guanine N-7 positions.	Interference with DNA damage repair processes and apoptosis induction.
Doxorubicin (**17**)	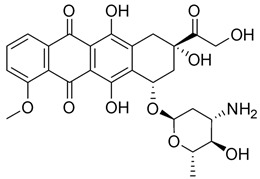	Intercalation and inhibition of DNA biosynthesis.	Inhibition of doxorubicin efflux from cancer cells and induction of apoptosis.
Cisplatin (**18**)	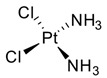	Binding to and interference with DNA transcription and replication.	Increase in CDDP toxicity and chromosome breakage.
Valproic acid (**19**)	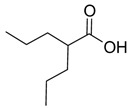	Blockage of voltage-gated ion channels and inhibition of histone deacetylase.	Apoptosis induction at lower concentrations with respect to monotherapy.
Taxol (**20**)	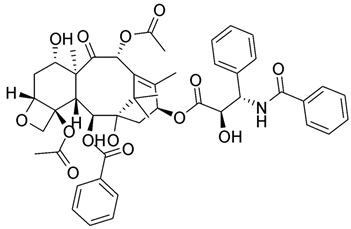	Stabilizing and preventing microtubules’ depolymerization, causing cell death and cell-cycle arrest at the G2/M phase.	Inhibition of cell growth and arrest, leading to the death of pancreatic adenocarcinoma cells.
Metformin (**21**)	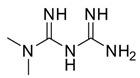	Alteration of the energy metabolism of the cell.	Inhibition of cell growth, an expansion of necrosis, and a reduction in tumor neo-vasculature.
5-Fluorouracil (**22**)	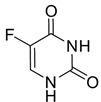	DNA damage via fraudulent incorporation in nucleic acid synthesis.	Inhibition of cell proliferation and induction of ROS-dependent apoptosis.

## Data Availability

Data sharing not applicable.
